# Coronary Computed Tomography Angiography Analysis of Calcium Content to Identify Non-culprit Vulnerable Plaques in Patients With Acute Coronary Syndrome

**DOI:** 10.3389/fcvm.2022.876730

**Published:** 2022-04-15

**Authors:** Théo Pezel, Georgios Sideris, Jean-Guillaume Dillinger, Damien Logeart, Stéphane Manzo-Silberman, Alain Cohen-Solal, Florence Beauvais, Niveditha Devasenapathy, Jean-Pierre Laissy, Patrick Henry

**Affiliations:** ^1^Department of Cardiology, Lariboisiere Hospital, Assistance Publique – Hôpitaux de Paris (APHP), University of Paris, Paris, France; ^2^Department of Radiology, Lariboisiere Hospital, Assistance Publique – Hôpitaux de Paris (APHP), University of Paris, Paris, France; ^3^Public Health Foundation of India, Indian Institute of Public Health, New Delhi, India

**Keywords:** coronary calcification, coronary computed tomographic angiography (CCTA), optical coherence tomography (OCT), non-ST elevation myocardial infarction (NSTEMI), vulnerable plaque

## Abstract

**Background:**

Aside from the culprit plaque, the presence of vulnerable plaques in patients with acute coronary syndrome (ACS) may be associated with future cardiac events. A link between calcification and plaque rupture has been previously described.

**Aim:**

To assess whether analysis of the calcium component of coronary plaques using CT angiography, coronary computed tomographic angiography (CCTA) can help to detect additional vulnerable plaques in patients with non-ST elevation myocardial infarction (NSTEMI).

**Materials And Methods:**

Cross sectional study of consecutive patients referred for NSTEMI from 30 July to 30 August 2018 with CCTA performed before coronary angiography with systematic optical coherence tomography (OCT) analysis of all coronary arteries within 24 h of clinical onset of NSTEMI. Three types of plaques were defined: culprit plaques defined by angiography (vulnerable culprit plaques–VCP) – plaques with a fibrous cap thickness < 65 microns or thrombus in OCT (vulnerable non-culprit plaque–VNCP) – plaques with a fibrous cap thickness ≥ 65 microns in OCT (stable plaque–SP).

**Results:**

A total of 134 calcified plaques were identified in 29 patients (73% male, 59 ± 14 years) with 29(22%) VCP, 28(21%) VNCP and 77(57%) SP. Using CCTA analysis of the calcium component, factors associated with vulnerable plaques were longer calcification length, larger calcification volume, lower calcium mass, higher Agatston score plaque-specific (ASp), presence of spotty calcifications and an intimal position in the wall. In multivariate analysis, ASp, calcification length and spotty calcifications were independently associated to vulnerable plaques. There was no difference between VCP and VNCP.

**Conclusions:**

CCTA analysis of calcium component of the plaque could help to identify additional vulnerable plaques in NSTEMI patients.

## Introduction

Acute coronary syndrome (ACS) remains a leading cause of mortality worldwide (1). The rupture of a thin-cap fibroatheroma (TCFA) leads to a coronary occlusion, which can lead to a myocardial infarction. Aside from this culprit plaque, one or several vulnerable plaques are most likely also present (2). Several decades of research on cardiovascular (CV) disease prevention have highlighted the importance of reducing the risk of recurrent ACS in patients with known coronary artery disease (CAD) (3). Recent studies have shown promising new therapy strategies to decrease the rate of recurrent CV events in these patients (4). Today, stratification of this risk can be achieved by a conventional analysis of available CV risk factors and by measuring high-sensitivity C-reactive protein levels (5). However, identifying the presence of other vulnerable plaques at an individual level remains challenging. Detailed analysis of plaque composition can be performed using intracoronary optical coherence tomography (OCT) with a very high resolution (10 μm). It has already been shown that vulnerable non-culprit plaques (VNCP) can be detected by OCT along with culprit plaques in patients with ACS (6). However, OCT is invasive and not widely available enough in clinical settings to be suitable for screening.

The coronary artery calcification score (CAC) is a well-known marker of the extent of atherosclerosis and is strongly associated with a high risk of adverse outcomes (7). However, the relationship between CAC and plaque vulnerability is complex. Some authors have claimed that the presence of calcification is associated with an increased likelihood of rupture (8), whereas others believe that the location and extent of calcification may, in fact, confer stability (9). Interestingly, it was reported that spotty calcifications (<3 mm diameter) are associated with a greater risk of ACS, suggesting again a role of CAC in plaque vulnerability (10)

Today, the overall CAC load of a patient can be assessed using calcium scoring on a non-injected computed tomography (CT) scan, as described by Agatston (11). A high Agatston score was strongly correlated with the risk of future CV events (12). We hypothesized that an analysis of the calcium content at the plaque level may be of interest to identify features that correlate with the presence of vulnerable plaques.

The aim of this study was to explore whether a detailed analysis of the calcium content of coronary calcified plaques using coronary computed tomographic angiography (CCTA) can help to predict the presence of other vulnerable plaques characterized by OCT in patients with ACS.

## Materials and Methods

### Study Population

From 30 July to 30 August 2018, we conducted a single-center cross-sectional study in patients with non-ST segment elevation myocardial infarction (NSTEMI) and an abnormal level of high-sensitivity cardiac troponin I (>50 ng/ml) (13). CCTA was performed to assess the presence of CAD. A coronary angiography was performed within the next 24 h only if a significant lesion (>50% stenosis) was detected by CCTA. An additional intracoronary OCT was systematically associated to check plaque erosion and other vulnerable plaques. Exclusion criteria were: (1) ST-segment elevation or a dynamic ECG change of repolarization; (2) recurrent chest pain; (3) hemodynamic instability with systolic blood pressure < 80 mmHg; (4) a history of bypass or coronary angioplasty; (5) renal failure with a glomerular filtration rate < 30 ml/min; (6) ventricular or supraventricular arrhythmia with a heart rate > 80 bpm; (7) a known allergy to the iodine contrast medium. All patients gave informed written consent, and the study was approved by the Ethics Committee of our institution.

### Coronary Computed Tomographic Angiography Data Acquisition and Analysis

CCTA was performed using an Aquilion Prime CT scanner (Toshiba Medical Systems, Otawara, Japan). The image acquisition protocol is detailed in Supplementary 1. Two experienced readers (JPL and TP) independently evaluated the reconstructed images while blinded to the clinical history and any prior findings.

Coronary calcification was defined as any extraluminal density >130 Hounsfield units (HU) over at least 4 adjacent contiguous sections of non-injected sites that could be assigned to the coronary arterial wall. The position and length of the plaques were defined manually by the radiologist along the arterial centerline. In line with prior studies (14), the intimal position of the calcification was defined by the presence of a calcification completely adjacent to the coronary lumen with no low-density area between the calcification and the lumen. Each plaque was analyzed using dedicated software (Vitrea^®^ 2.1, Vital Images Inc., Minnetonka, MN, United States) to identify risk factors of vulnerable plaque according to SCCT guidelines (15, 16). Details of the methods for using this dedicated software are described in Supplementary 2. The contours of the plaque were edited by two experienced radiologists when necessary. The radiologist selected the start and end of a plaque, and the following parameters were obtained automatically: the plaque and calcification lengths, the plaque and calcification volumes, the minimum and maximum calcification diameters, the area of stenosis, the Agatston score (plaque specific; ASp) for each plaque, the sphericity index (SI) of calcification in the axial section, the plaque burden, and the remodeling index (RI). ASp corresponds to the Agatston calcium score measured for each plaque, using a semi-automated tool to calculate the CAC score based on the extent of coronary artery calcification as previously described (11). Of note, each calcified plaque was defined by a structure with a CT number from – 50 to 750 HU adjacent to the lumen including a joined set of at least 4 adjacent contiguous sections with density > 130 Hounsfield units (HU). The contours of each plaque were edited by two experienced radiologists. Plaques with spotty calcifications were defined as follows: length (extent in the longitudinal direction of the vessel) of the calcification < 3/2 of vessel diameter and width (extent of the calcification perpendicular to the longitudinal direction of the vessel) of the calcification < 2/3 of vessel diameter, as previously described (17). The reproducibility data of the calcification measurements was evaluated with good to excellent intra- and inter-reader reproducibility (intraclass correlation ranging from 0.77 to 0.97; all *p* < 0.001). Details regarding the data analysis protocol and radiation exposure are provided in Supplementary 1 and 3, respectively.

### Angio and Optical Coherence Tomography Image Acquisition and Analysis

**FIGURE 1:**
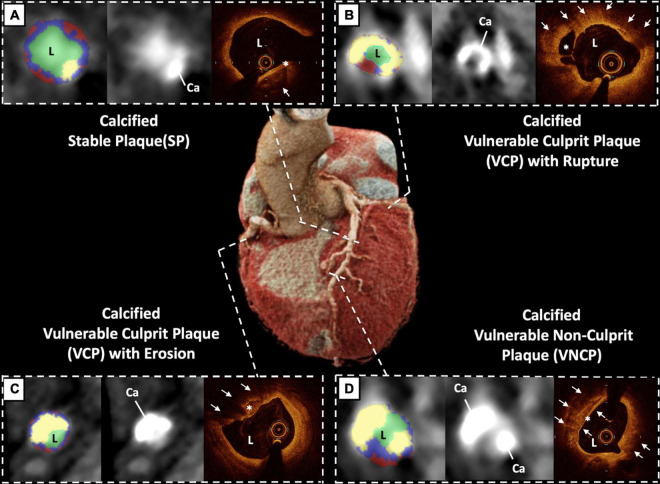
Example of three different coronary calcified plaques studied by coronary computed tomographic angiography (CCTA) with matching optical coherence tomography (OCT) and “virtual histology” CCTA in the same patient. **(A)** stable plaque (SP): adventitious calcification (white arrow) with a thick fibrous screed ≥ 65 microns (*) at the mean LAD level. **(B)** vulnerable culprit plaque (VCP) with plaque rupture: large intimal quasi-circumferential calcification with a calcification arc at 240° (white arrows) with rupture of white thrombotic plaque (*) at the level of the proximal circumflex artery. **(C)** VCP with plaque erosion: large intimal calcification bulging toward the light (white arrows) with disappearance of the intima opposite (*) at the level of the proximal right coronary artery. **(D)** vulnerable non-culprit plaque (VNCP): intimal calcification (white arrows) with a fine fibrous screed < 65 microns (*) at the distal left anterior descending artery. *Abbreviations:* Ca, Calcification; CCTA, coronary computed tomographic angiography; L, Lumen; OCT, optical coherence tomography; SP, stable plaque; VNCP, vulnerable non-culprit plaque; VCP, vulnerable culprit plaque.

Angio, with the aid of electrocardiogram or echo data if needed, was used to define culprit plaque (vulnerable culprit plaque: VCP). Intracoronary OCT (Ilumien Optis OCT, St. Jude Medical, St. Paul, MN, United States) was performed before the percutaneous coronary intervention (PCI). All coronary arteries were systematically analyzed with OCT. The OCT image analysis was performed by two experienced cardiologists (GS and JGD), blinded to clinical and CCTA data, using previously established criteria for OCT plaque characterization (18). The segmentation between vulnerable and stable plaques was carried out on the TCFA and/or the thrombus. The hempen cap thickness (FCT) was measured 3 times at its thinnest part, and the average value was calculated. A VNCP was defined as a lipid-rich plaque with a cap thickness < 65 μm or the presence of thrombus. Stable plaque (SP) was defined as a cap thickness ≥ 65 μm without any thrombus (Figure 1). A detailed protocol of OCT image acquisition and analysis can be found in Supplementary 4.

### Statistical Analysis

Continuous variables are presented as mean ± standard deviation (SD) and categories as percentages ± SD. Comparisons of the plaque characteristics were performed using the Mann–Whitney test or Fisher’s exact test. Intraclass correlation coefficients were calculated to assess intra-observer and inter-observer agreement for the lipid arcs, and kappa statistics were used to assess the agreement of the plaque characterizations.

Based on a linear regression model, the vulnerability of the plaque was established. OCT indices for plaque vulnerability were assessed, such as the presence of TCFA, plaque rupture, macrophage accumulations, a microchannel, intracoronary thrombus, and FCT. A multivariable analysis was performed to identify covariates obtained from CCTA that was significantly associated with vulnerable plaque, defined by OCT. This multivariable stepwise logistic regression analysis including the following covariables: age, male gender, diabetes mellitus, chronic kidney disease, ASp, calcium length, and the presence of spotty calcifications. The model fit was assessed using C-index and area under the receiver operating characteristic (ROC) curves. All the statistical tests were two-sided, and *p*-values < 0.05 were considered statistically significant. The statistical analysis was performed using GraphPad Prism, v7 (GraphPad Software Inc., San Diego, CA, United States) and Stata SE v12.1 (Statacorp, Texas, United States).

## Results

### Study Population and Characterization of Plaques

**FIGURE 2:**
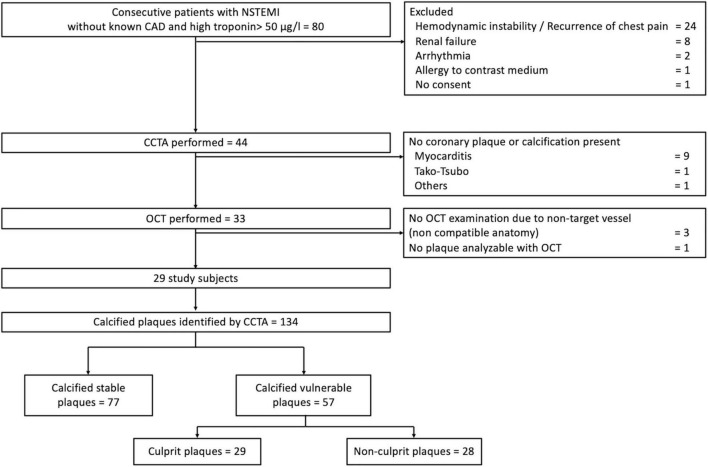
Study flowchart. *Abbreviations:* ACS, acute coronary syndrome; CAD, coronary artery disease; CCTA, coronary computed tomographic angiography; FCT, fibrous cap thickness; NSTEMI, non–ST-segment elevation myocardial infarction; OCT, optical coherence tomography.

The flowchart of study participants is given in Figure 2, resulting in a total of 134 calcified plaques analyzed. The clinical characteristics and the laboratory data of the subjects are summarized in Table 1. Of the 134 coronary calcified plaques identified by CCTA, there were 29 (22%) VCP. After the OCT analysis, we found 28 (21%) VNCP and 77 (57%) SP.

**TABLE 1:** Baseline characteristics of the patients.

	*N* = 29 patients
Age, yrs	59.1 ± 13.5
Male, *n* (%)	21 (72.5)
BMI, kg/m^2^	26.7 ± 4.2
Coronary risk factors, *n* (%)	
DM	4 (13.8)
Hypertension	15 (51.7)
Dyslipidemia	11 (37.9)
Current smoker	16 (55.2)
Family history	8 (27.6)
**Laboratory data**	
Peak hs-TnI, μg/l	1,343 ± 2891
Peak CPK, IU/l	175 ± 162
LDL-c, mg/dl	135.1 ± 43.2
HDL-c, mg/dl	43.3 ± 12.1
TG, mg/dl	142 ± 80
HbA1c,%	6.3 ± 1.2
hs-CRP, mg/dl	8.0 ± 10.7
NT-pro-BNP, pg/mL	408.9 ± 536.7
Creatinine clearance CKD-EPI, ml/min/1.73 m^2^	91.1 ± 15.3
GRACE score	108.1 ± 27.4
**Medications before ACS, *n* (%)**	
Aspirin	8 (27.6)
β-Blockers	5 (17.2)
Statins	4 (13.8)
ACE-I/ARBs	8 (27.6)
Medications before ACS, *n* (%)	
Aspirin	29 (100)
Ticagrelor, Clopidogrel, or Prasugrel	27 (93.1)
LVEF,%	55.3 ± 9.0
**CT scan data**	
Heart rate, bpm	65 ± 12
Number of calcified plaques per patient	4.4 ± 2.8 [1–11]
Number of vulnerable plaques per patient	2.1 ± 1.9
Number of stable plaques per patient	2.8 ± 2.1
Agatston score	287 ± 294 [12–325]

*The values are means ± the SD or n (%). The creatinine clearance rate was calculated using the MDRD formula.*

The characteristics of the coronary plaques in OCT are summarized in Table 2.

**TABLE 2:** Comparison of calcified plaques depending on the vulnerability profile by optical coherence tomography (OCT).

	VNCP (*N* = 28)	VCP (*N* = 29)	*p*- value*	SP (*N* = 77)	*p*-value**
Lipid-rich plaque, *n* (%)	18 (67)	20 (69)	0.96	32 (47)	<0.0001
Lipid length, mm	4.3 ± 4.5	4.4 ± 4.9	0.95	1.0 ± 2.8	<0.0001
Maximum lipid arc,°	121.9 ± 107.7	113.5 ± 114.2	0.99	21.3 ± 52.6	<0.0001
Minimum fibrous cap thickness, μm	19.6 ± 20.4	22.6 ± 47.9	0.10	143.5 ± 59.6	<0.0001
TCFA, *n* (%)	25 (96)	27 (93)	0.99	0 (0)	<0.0001
Plaque rupture, *n* (%)	0 (0)	21 (72)	<0.0001	0 (0)	0.99
Plaque erosion, *n* (%)	0 (0)	5 (16)	<0.0001	0 (0)	0.99
Thrombus, *n* (%)					
Red thrombus	0 (0)	1 (3)	0.08	0 (0)	0.99
White thrombus	0 (0)	26 (91)	<0.0001	0 (0)	0.99
Healed plaque rupture, *n* (%)	1 (2)	0 (0)	0.08	0 (0)	0.99
Cholesterol crystal, *n* (%)	3 (6)	2 (6)	0.08	1 (2)	0.04
Microchannel, *n* (%)	1 (2)	3 (9)	0.09	0 (0)	0.03
Macrophage accumulation, *n* (%)	4 (8)	2 (6)	0.07	0 (0)	0.02
Area stenosis,%	67.7 ± 8.6	78.2 ± 8.9	0.01	47.7 ± 7.2	0.01
Calcification length, mm	6.6 ± 4.3	6.7 ± 5.1	0.90	2.9 ± 2.1	<0.001
Maximum calcification arc,°	136.1 ± 96.4	139.7 ± 100.7	0.80	55.4 ± 45.9	<0.0001

*Comparison between calcified stable plaques and calcified vulnerable plaques. **Comparison between calcified stable plaques and vulnerable plaques without culprit lesion. The values are means ± the SD or n (%). SD, standard deviation; SP, stable plaque; TCFA, thin-cap fibroatheroma; VCP, vulnerable culprit plaque; VNCP, vulnerable non-culprit plaque.

### Coronary Computed Tomographic Angiography Characteristics of Calcified Plaques

The calcified plaque variables examined by CCTA to distinguish VNCP, VCP, and SP are shown in Table 3. The data are provided separately for the entire plaque and for the calcium content of the plaque. There was no significant difference between VNCP and VCP except for the maximum diameter of calcification (*p* = 0.01) while there were many significant differences between VNCP and SP. Indeed, the VNCPs were significantly longer (*p* < 0.001), with a larger total volume (*p* < 0.001), higher plaque burdens (*p* < 0.001), a finer degree of stenosis (*p* < 0.001), and a greater percentage of low-density plaque content (< 30 or < 50 HU) compared to SP (*p* < 0.001).

**TABLE 3:** Univariate analysis of the comparison of calcified plaques depending on the vulnerability profile by CCTA (*N* = 134).

	VNCP (*N* = 28)	VCP (*N* = 29)	*p*-value*	SP (*N* = 77)	*p*-value**
Entire plaque					
*Plaque length, mm*	7.1 ± 4.2	8.2 ± 5.9	0.73	4.0 ± 3.0	<0.0001
Plaque volume, mm^3^	141.8 ± 88.1	178.7 ± 139.5	0.09	72.9 ± 56.8	<0.0001
Plaque burden,%†	69.4 ± 10.1	73.2 ± 17.2	0.15	59.4 ± 12.0	<0.0001
Min lumen area, mm^2^	4.6 ± 2.7	2.7 ± 3.0	0004	6.1 ± 3.3	0.03
Arterial remodeling index††	1.16 ± 0.19	1.20 ± 0.16	0.60	1.02 ± 0.15	0.001
Distance from aorta, mm‡	25.4 ± 14.9	21.8 ± 6.1	0.77	29.1 ± 12.2	0.11
Mean plaque density, HU	267.4 ± 78.4	250.7 ± 72.0	0.35	265.9 ± 80.3	0.94
Plaque < 30HU, mm^3^	16.6 ± 8.9	21.1 ± 22.7	0.44	4.1 ± 3.5	<0.0001
Plaque < 30HU,%	13.3 ± 5.4	12.4 ± 6.1	0.73	6.8 ± 4.5	<0.0001
Plaque < 50HU, mm^3^	25.7 ± 16.6	35.0 ± 31.7	0.41	8.1 ± 7.2	<0.0001
Plaque < 50HU,%	21.0 ± 10.1	22.1 ± 11.6	0.68	11.9 ± 5.7	<0.0001
LAP, < 50HU ≥ 10% of total plaque, *n (%)*	23 (82.1)	21 (72.4)	0.49	53 (68.8)	0.18
LAP, < 50HU ≥ 20% of total plaque, *n (%)*	15 (53.6)	18 (62.1)	0.52	5 (6.5)	<0.0001
Plaque < 150 HU, mm^3^	52.5 ± 40.2	68.9 ± 51.6	0.21	37.6 ± 33.8	0.03
Plaque < 150 HU,%	42.9 ± 25.2	51.0 ± 27.0	0.33	53.5 ± 23.0	0.04
Calcium component > 355 HU,%	57.1 ± 25.2	49.0 ± 27.0	0.34	46.4 ± 23.0	0.04
Plaque-artery relationship					
Stenosis,%	47.6 ± 25.2	73.1 ± 27.1	<0.0001	25.9 ± 20.7	<0.0001
Plaue facing					
-myocardium, *n* (%)**§**	11 (39.3)	6 (20.7)		32 (41.6)	0.32
-pericardium, *n* (%)**§**	12 (42.9)	11 (37.9)	0.16	39 (50.6)	
-myocardium and pericardium, *n* (%)	5 (62.5)	12 (41.4)		6 (7.8)	
Plaque includes					
-inner curve of artery, *n (%)*	15(53.6)	8(27.6)		32(41.6)	0.08
-outer curve of artery, *n (%)*	10 (35.7)	15 (51.7)	0.17	43 (55.8)	
-both inner and outer curves, *n (%)*	3 (10.7)	6 (29.7)		2 (2.6)	
True bifurcation (vs. all other plaques), *n* (%) **#**	6 (21.4)	6 (20.7)	0.98	5 (6.)	0.03
Calcium component of the plaque					
Calcification length, mm	6.2 ± 4.5	7.0 ± 5.8	0.92	3.0 ± 2.0	<0.0001
Calcification volume, mm^3^	89.3 ± 75.1	109.7 ± 120.4	0.95	35.3 ± 40.8	<0.0001
Calcium mass, mg	15.7 ± 14.1	18.7 ± 22.8	0.85	7.5 ± 12.	0.003
Calcium mass/volume ratio, mg/mm^3^	0.16 ± 0.05	0.16 ± 0.05	0.85	0.20 ± 0.10	0.08
Mean calcification density, HU	483.7 ± 114.1	464.0 ± 101.6	0.57	519.7 ± 140.1	0.17
ASp	111.4 ± 90.8	138.9 ± 148.0	0.77	32.7 ± 37.5	<0.0001
Distance lumen to calcification, mm	1.06 ± 0.34	0.95 ± 0.37	0.19	1.43 ± 0.41	<0.0001
Minimal diameter of calcification, mm	2.0 ± 0.6	1.7 ± 0.7	0.06	2.3 ± 0.7	0.08
Maximal diameter of calcification, mm	3.1 ± 0.7	2.6 ± 0.8	0.01	3.1 ± 0.8	0.86
Sphericity index	0.65 ± 0.11	0.66 ± 0.16	0.95	0.74 ± 0.13	0.001
Presence of spotty calcification, *n (%)*	13 (46)	15 (51.7)	0.68	12 (16)	0.001
Napkin-ring sign, *n (%)*	3 (10.7)	2 (6.9)	0.52	4 (5.2)	0.32

The values are means ± the SD or n (%). *Comparison between vulnerable plaques without culprit lesion and calcified stable plaques. **Comparison between vulnerable plaques without culprit lesion and culprit plaques. **†**Calculated as the percentage plaque volume/total arterial volume along the length of the plaque. **‡**Measured to the proximal border of the plaque. **§** At least part of the plaque facing the myocardium or pericardium, respectively. **#**Medina type 3 (plaque proximal, directly opposite, and distal to a side branch) versus all others. textbf†⁣†Cross-sectional area of the artery at the maximal plaque area/proximal arterial reference area.

**FIGURE 3:**
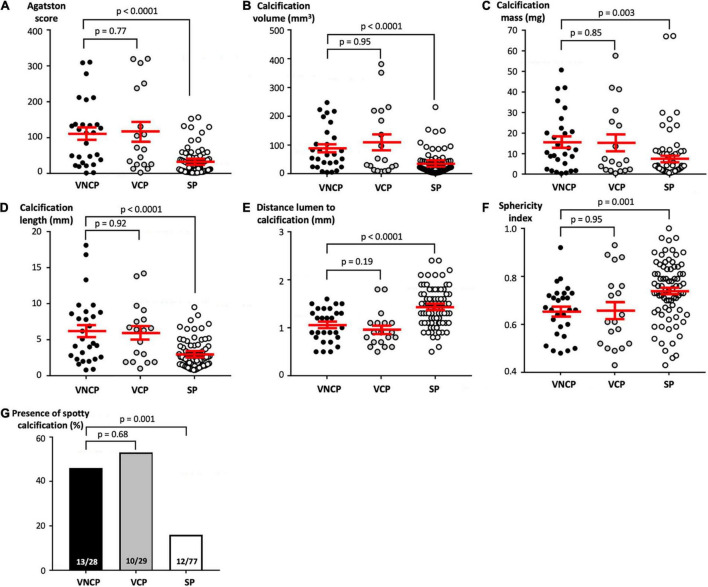
Dot-plots comparing CCTA characteristics of VNCP, VCP, and SP. Comparisons of the Agatston score per calcified plaque **(A)**, the calcification volume in mm^3^ **(B)**, the calcification masses in mg **(C)**, the calcification length in a longitudinal section in mm **(D)**, the calcification position on the wall measured by the distance in mm between the most intimal part of calcification and the vessel lumen **(E)**, the sphericity index defined as the ratio between the minimum diameter and the maximum diameter in an axial section **(F)**, and the presence of spotty calcification (percentage; **G**). Quantitative variables are expressed as mean ± SEM. *Abbreviations:* CCTA, coronary computed tomographic angiography; SEM, standard error of the mean; SP, stable plaque; VNCP, vulnerable non-culprit plaque; VCP, vulnerable culprit plaque.

When focusing on the calcium content of the plaques, compared to SP, VNCP had longer calcification lengths (*p* < 0.001), a larger volume of calcification (*p* < 0.001), a lower mass of calcium (*p* = 0.003), a higher ASp (*p* < 0.001), a lower SI (*p* = 0.001), more spotty calcifications (*p* = 0.001), and a more intimal position on the coronary vessel wall, defined by a shorter distance between the innermost part of the calcification and the lumen (*p* < 0.001; Figure 3).

### Estimation of the Risk of Plaque Vulnerability Based on Multivariate Analysis

**FIGURE 4:**
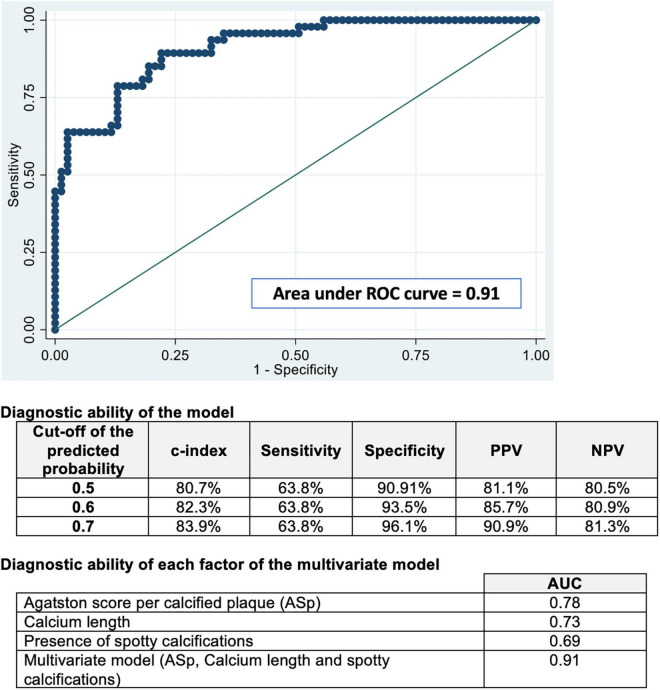
ROC curve analysis for the detection of vulnerable plaques by the Agatston score plaque-specific, calcium length, and the presence of spotty calcifications. The receiver-operating characteristics (ROC) curve, area under the ROC curve, and Hosmer-Lemeshow goodness-of-fit tests for our model to predict the plaque vulnerability defined by OCT. Our model takes into account the Agatston score plaque-specific (ASp), the calcium length, and the presence of spotty calcifications, in addition to age and gender (AUC = 0.91). *Abbreviations:* AUC, area under the curve; ASp, Agatston score per calcified plaque; NPV, negative predictive value; PPV, positive predictive value; ROC, receiver operating characteristics.

In the multivariate model (including gender and age) to identify vulnerable plaques, ASp, calcium length, and the presence of spotty calcifications were significantly associated with the presence of vulnerable plaques. The area under the ROC curve for the identification of vulnerable plaque was 0.91 (Hosmer–Lemeshow goodness of fit *p* = 0.76) for the model containing ASp, calcium length, and the presence of spotty calcifications (Figure 4). There was no evidence of multicollinearity (the overall variance inflation factor of the model was 2.78).

## Discussion

**FIGURE 5:**
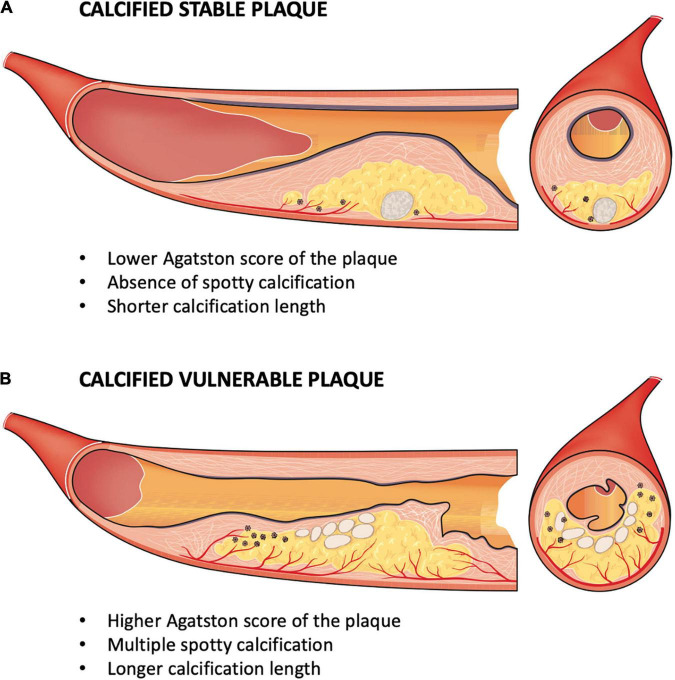
Physical and geometric model of the calcium component of vulnerable plaques. **(A)** Calcified stable plaque had a smaller total calcium volume, a lower density (HU), a lower Agatston score of the plaque (ASp; longitudinal section – to the left); a more eccentric position and a higher sphericity index (axial section – to the right). **(B)** Calcified vulnerable plaque had a larger total calcium volume, a higher density (HU), a higher ASp and multiple spotty calcifications (longitudinal section – to the left); a more intimal position leading to mechanical stress and with a smaller sphericity index, corresponding to more oval calcifications (axial section – to the right). *Abbreviations:* Asp, Agatston score per calcified plaque; HU, Hounsfield unit.

In the present study, we showed that an analysis of the calcium content of a plaque using CCTA can help to identify VNCP other than VCP. Our results show that vulnerable plaques had a higher ASp, a longer calcification length, and multiple spotty calcifications compared to the SPs in the multivariate analysis. Moreover, we studied a model based on these three parameters, which shows an excellent predictive value for the detection of VCP (Figure 5).

### Prognostic Value of Coronary Computed Tomographic Angiography

This potential prognostic value of CCTA to predict the risk of ACS with vulnerable plaque and, therefore, the recurrence of ACS, has been described in several studies. Low attenuation plaque (< 30 HU), the napkin-ring sign, and positive remodeling on CCTA were associated with the development of ACS during medium-term (a mean of 3.9 years) follow-up (19). Previous studies also found that low attenuation plaque, positive remodeling, and a napkin-ring sign using CCTA were associated with a TCFA with OCT in a per-lesion-level analysis (20). Indeed, Montone et al. have recently described the importance of intracoronary imaging for the management of non-culprit lesion in ACS to identify vulnerable plaques that may portend a higher risk of future cardiovascular events (21).

A recent study comprising 3,646 patients with known CAD and a median follow-up of 5 years revealed the prognostic value of CCTA, which is associated with higher rates of all-cause mortality and major adverse CV events at 5 years (22). Our results regarding the analysis of calcified plaques confirm these results for low attenuation plaque and positive remodeling, and they show that the volume and burden of the plaque were associated with plaque vulnerability.

### Calcification and Plaque Vulnerability

Several studies using cardiac CT have reported that the total CAC score is associated with a high risk of adverse CV outcomes (12). Interestingly, the risk of CAD and long-term cardiovascular events is extremely low with a CAC score of zero. All these findings show that coronary calcification plays an important role in the risk stratification of CAD (23). However, it is still unclear how calcification affects the stability of plaques as some authors have claimed that the presence of calcification is associated with an increased likelihood of rupture (8, 24), whereas others believe that the location and extent of calcification may, in fact, confer stability (9). It has been suggested that calcification within the lipid core, away from the hempen cap (as opposed to within or in close proximity to the cap), may stabilize the plaque (9).

In a recent review, Henein M et al. described that coronary calcification develops as an immune response to endothelial injury, such as shear stress or oxidative stress in diabetics, and is consequently part of the body’s natural defenses (25). Although it is a systemic condition, no medical treatment for cardiovascular disease has yet found a way to regress these calcifications; on the contrary, lipid-lowering agents may worsen its progression (25). Indeed, several studies show an increase in coronary calcifications in patients treated with statins (20, 21).

While extensive plaque calcification is often a feature of advanced and stable atherosclerosis, which rarely leads to rupture, several studies report that microcalcifications are strongly correlated with plaque vulnerability and the risk of incident ACS in the medium and long term. Indeed, microcalcifications develop in the early stages of coronary intimal calcification and contribute directly to plaque rupture by producing local mechanical stress on the plaque surface (26). Microcalcification is known to occur in TCFA, which is not visible using current *in vivo* imaging methods. Indeed, inflammation has a key role in atherosclerotic plaque formation and rupture. Intense macrophage inflammatory activity results in microcalcifications which are strongly associated with plaque vulnerability (27). The pioneering work of Vengrenyuk (28) attributed cap rupture to microcalcification within the cap (29). It is believed that microcalcifications act as local tissue stress concentrators within the fibrous cap, thereby predisposing it to rupture (30). Although some ruptures are associated with cap microcalcification, this is not a consistent finding with plaque ruptures (31). Therefore, this potential role of calcification in the vulnerability of plaques was described with calcification nodules that may extend into the lumen or the media and can be associated with fibrin deposition. These protuberant calcifications can lead to the discontinuity of the endothelial lining and underlying collagen matrix as well as acute luminal thrombosis. Calcified nodules are the underlying mechanism of ACS in 2–7% of coronary artery thromboses (32).

A recent clinical CT study that examined the predictive value of both calcium density (standard CAC Agatston score) and plaque calcium density showed that although the CAC score was positively and independently associated with CV disease risk, at any level of CAC volume, CAC density was inversely and significantly associated with CV disease risk (12, 33), as we found in our study. In addition, a recent study showed that a CAC score of zero at baseline in an asymptomatic population with no known CV disease was associated with low CAC progression over the 5-year period. This study also described that the use of the CAC score improved CV risk stratification and reclassification in this primary prevention population (34). All these studies have led the Society of Cardiovascular Computed Tomography (SCCT) guidelines committee has produced new guidelines to guide the use of CCTA in the risk assessment of CAD in occupational health evaluation (16).

Interestingly, we showed the relevance of a model using ASp, calcium length, and the presence of spotty calcifications to detect vulnerable plaque. These results are consistent with a study that showed that the ASp in the ACS group was higher than in the asymptomatic group and similar to the stable angina pectoris group. Moreover, the calcified plaques in the ACS group exhibited lower and more homogenous attenuation than those of the asymptomatic or stable angina pectoris group (33).

### The Mechanical Role of Calcification in Vulnerability

Our study demonstrates that, apart from analyzing spotty calcifications, an analysis of the calcium content of plaques using CCTA can help with the detection of vulnerable plaques. Indeed, a relatively large intraplaque calcification volume but a low calcium density may correspond at least to a less structurally stable calcification. This may explain the role of calcification in the destabilization of a coronary plaque with unstable calcification. Moreover, a more intimal position near the lumen is an additional element that suggests a possible mechanical role of calcification in the vulnerability of a plaque by mechanical shear stress on the fibrous cap. These results favor a physical and geometric model of the role of the calcium content of plaque as a vulnerability factor (Figure 5).

### Study Limitations

The study has some limitations. First, this cross-sectional single-center study only assessed a limited number of patients because a systematic OCT analysis of the entire coronary bed was performed. However, 134 plaques were fully analyzed simultaneously by CCTA and OCT. Second, since the population of this study only included patients referred for NSTEMI, the results of this study can only be applied to NSTEMI patients. Third, we applied semiautomatic methods to perform the plaque quantification using CCTA because contour drawing or manual confirmation in a semiautomatic analysis by an expert reader is more accurate than a fully automatic method (35). Even though there have been several studies regarding plaque quantification using CCTA, there is still a need for further refinements in terms of the reproducibility, accuracy, and standardization of the analysis (36). Fourth, although OCT is an innovative intravascular imaging method with a high spatial resolution (10–15μm) (37), it may not be the perfect reference standard for detecting vulnerable plaques. However, even if histology is usually considered as the gold standard for defining plaque vulnerability, OCT is clearly of considerable value as many studies have shown its prognostic value to predict clinical events (6, 38). Furthermore, we only studied patients at the time of their first ACS, without known prior CAD, to avoid any confounding factors related to differences in medical or interventional treatments prior to the ACS. Fifth, this study did not compare hypodense plaques with calcified plaques in CCTA, as has been done previously (39). Indeed, we decided to focus solely on the analysis of calcified plaques because recent studies suggest a crucial role of calcification in plaque vulnerability. Finally, our study used a surrogate parameter, which is vulnerable plaques defined by OCT. This quantitative parameter has the advantage of being robust and reproducible, with many studies showing its association with the risk of CV events, including the recurrence of ACS in patients with known CAD (6).

## Conclusion

Our study of NSTEMI patients shows that an analysis of the calcium content of plaques using CCTA could help to detect VNCP. In the CCTA analysis, vulnerable plaques had a higher ASp, a longer calcification length, and multiple spotty calcifications compared to SPs, with a high predictive value.

## Data Availability Statement

The raw data supporting the conclusions of this article will be made available by the authors, without undue reservation.

## Ethics Statement

The studies involving human participants were reviewed and approved by CPP. The patients/participants provided their written informed consent to participate in this study.

## Author Contributions

All authors participated in the discussion of the concept of the study. TP and PH conceived the study design. TP and J-PL obtained CCTA images and analyzed CCTA scans. TP, PH, GS, J-GD, SM-S, and DL obtained OCT images, and TP and GS analyzed OCT scans. TP and ND performed statistical analyses. TP and PH analyzed data and drafted the manuscript with critical revision. As authors, we attest to each of our substantial contributions to the manuscript and revision. All authors read and approved the final manuscript.

## Conflict of Interest

The authors declare that the research was conducted in the absence of any commercial or financial relationships that could be construed as a potential conflict of interest.

## Publisher’s Note

All claims expressed in this article are solely those of the authors and do not necessarily represent those of their affiliated organizations, or those of the publisher, the editors and the reviewers. Any product that may be evaluated in this article, or claim that may be made by its manufacturer, is not guaranteed or endorsed by the publisher.

## Abbreviations

ACS: acute coronary syndrome
ASp: Agatston score (plaque specific)
CAC: coronary artery calcification
CAD: coronary artery disease
CCTA: coronary computed tomographic angiography
CT: computed tomography
CV: cardiovascular
HU: Hounsfield unit
NSTEMI: non-ST segment elevation myocardial infarction
OCT: optical coherence tomography
SD: standard deviation
SP: stable plaque
TCFA: thin-cap fibroatheroma
VCP: vulnerable culprit plaque
VNCP: vulnerable non-culprit plaque.
