# Supplementary material for: Coronary Computed Tomography Angiography Analysis of Calcium Content to Identify Non-culprit Vulnerable Plaques in Patients With Acute Coronary Syndrome
Source: Front Cardiovasc Med. 2022 Apr 15;9:876730. doi: 10.3389/fcvm.2022.876730 (PMC9051337; doi:10.3389/fcvm.2022.876730)
Supplement: Supplementary file 1 [file Data_Sheet_1.pdf]

SUPPLEMENTS

Table of Contents:

- Supplement 1. Patient Preparation Before CCTA and CCTA Data Analysis.
- Supplement 2. CCTA data using the dedicated software (Vitrea®).
- Supplement 3. Radiation Exposure.
- Supplement 4. OCT Image Acquisition and OCT Data Analysis.

SUPPLEMENT 1

PATIENT PREPARATION BEFORE CCTA

The patients were continuously monitored by ECG and defibrillator during transport to the CCTA and during the entire examination until the percutaneous coronary intervention (PCI) was performed. Prior to the CCTA, in patients with a resting heart rate (HR) > 65 beats/min, atenolol was administered intravenously, with a titrated dose of up to 50 mg to achieve a target HR of ≤ 65 beats/min. All of the patients received sublingual nitrates to ensure coronary vasodilation before the rest scan.

CCTA DATA ANALYSIS

All of the scans were examined in axial, multiplanar reformat, and short-axis cross-sectional views. For the CCTA analysis, the coronary arteries were segmented as recommended by the American Heart Association (AHA).(1)

Two cardiac imagers who were blinded to the clinical history and the invasive evaluation findings independently evaluated the reconstructed images.(2)

The window settings were adjusted by the operator to obtain the best differentiation between plaque, surrounding tissue, and vessel lumen and to differentiate between intraplaque densities. Calcified plaque was defined as any extraluminal density that could be clearly assigned to the coronary arterial wall with coronary calcification. Coronary calcification was classically defined as any extraluminal density > 130 HU over at least 4 adjacent contiguous sections of non-injected sites that could be assigned to the coronary arterial wall.(3) The plaque position and the length were defined along the arterial centerline. Dedicated cardiac analysis software with a plaque analysis application (Vitrea® 2.1, Vital Images Inc., Minnetonka, Minnesota, USA) was used to analyze each plaque separately for plaque definition and analysis with manual adjustment as required.

A structure with a CT number from – 50 to 750 HU adjacent to the lumen was identified as a plaque. The contours of the plaque were edited by two experienced radiologists when necessary. The radiologists selected the start and the end of a plaque, and the following parameters were obtained automatically(4): the plaque and the calcification length, the plaque and the calcification volume, the minimum and the maximum calcification diameter, the area of stenosis, the Agatston score plaque-specific (ASp) corresponding to the Agatston calcium score for each plaque, the Sphericity Index (SI) of calcification in the axial section, the plaque burden, and the remodeling index (RI). Although other methods of scoring have been used, the Agatston

score(3) is the gold standard as a result of its simplicity. The SI was defined as the ratio between the minimum diameter and the maximum diameter in the axial section, corresponding to evaluation of the circumferential extension of calcification. The plaque burden was calculated for each plaque as the volume of the plaque divided by the total volume of the same section of coronary artery containing the plaque.(4,5) The RI was measured as the maximal cross-sectional artery area at the plaque/plaque-free cross-sectional area, located proximally whenever possible.(5) Positive remodeling (PR) was defined by an RI > 1.1.(4) The location of intimal, medial, or adventitial calcification of the coronary wall was evaluated both qualitatively upon visual inspection by the operators, as well as by the distance between the center of the vessel lumen and the innermost part of the calcification. A graphic representation of the frequency distribution of the volume and the CCTA density of each constituent plaque was provided by the software and stored numerically in a data file. To exclude artifacts at arterial borders, CCTA densities below –40 Hounsfield units (HU) were considered to be fatty tissue outside the arterial wall and were hence excluded from the analysis. Percentages of plaque volume with < 30 HU, < 50 HU, < 150 HU, and between 351-1,000 HU were calculated.(5) The minimum CCTA number of the plaque was also measured using several circular regions of interest (areas of 1 mm^2) at each plaque site. In order to assess the tissue density of the calcification, measurement of the calcium mass was carried out to obtain the density of the calcification defined by the ratio of the calcium mass and the volume of the calcification, associated with the measurement of the calcification average density of calcification. Presence of the napkin-ring sign was defined as a plaque core with low-attenuation surrounded by a rim-like area of higher attenuation, but not exceeding 130 HU.(4)

Patients with ACS underwent invasive angiography at the presenting hospital. The culprit plaque was identified on the angiographic image with the aid of clinical data as necessary and on the baseline CCTA with the aid of anatomical landmarks.

Interobserver variation was examined in 45 individual coronary plaques in 10 patients following resegmentation, to assess the correlation.

References

1. Cerqueira MD, Weissman NJ, Dilsizian V, Jacobs AK, Kaul S, Laskey WK, et al. Standardized myocardial segmentation and nomenclature for tomographic imaging of the heart. A statement for healthcare professionals from the Cardiac Imaging Committee of the Council on Clinical Cardiology of the American Heart Association. Circulation. 2002;105(4):539–42.

2. Austen WG, Edwards JE, Frye RL, Gensini GG, Gott VL, Griffith LS, et al. A reporting system on patients evaluated for coronary artery disease. Report of the Ad Hoc Committee for Grading of Coronary Artery Disease, Council on Cardiovascular Surgery, American Heart Association. Circulation. 1975;51(4 Suppl):5–40.

3. Agatston AS, Janowitz WR, Hildner FJ, Zusmer NR, Viamonte M, Detrano R. Quantification of coronary artery calcium using ultrafast computed tomography. J Am Coll Cardiol. 1990;15(4):827–32.

4. Chun EJ, Han JH, Yoo SM, Lee HY, Song IS, White CS. Differences in the CT findings between vulnerable plaque and culprit lesions in acute coronary syndrome. J Cardiovasc Comput Tomogr. 2018;12(2):115–7.

5. Yang DH, Kang S-J, Koo HJ, Chang M, Kang J-W, Lim T-H, et al. Coronary CT angiography characteristics of OCT-defined thin-cap fibroatheroma: a section-to-section comparison study. Eur Radiol. 2018;28(2):833–43.

SUPPLEMENT 2

CCTA DATA USING THE DEDICATED SOFTWARE (VITREA®)

CT SUREPlaque is a specific function of the dedicated software (Vitrea® 2.1, Vital Images Inc., Minnetonka, MN, USA) to assess each coronary plaque. It provides the visualization and measurement of vessel walls and plaque characteristics in arterial vessels using color defined Hounsfield Unit (HU) ranges through a streamlined workflow.

CT SUREPlaque tools assist in evaluating the characteristics inside blood vessels:

- Quantify plaque burden and coronary remodeling non-invasively
- Visualize coronary vessel anatomy and disease with ease using defined HU ranges
- Characterize a lesion in the vessel wall as either calcified or non-calcified
- Single-click segmentation with automatic centerline and lumen boundaries
- Automatic measurement and display of: lumen area and diameter; plaque area; plaque burden; ratio of wall area and lumen area; plaque volume; and plaque index.

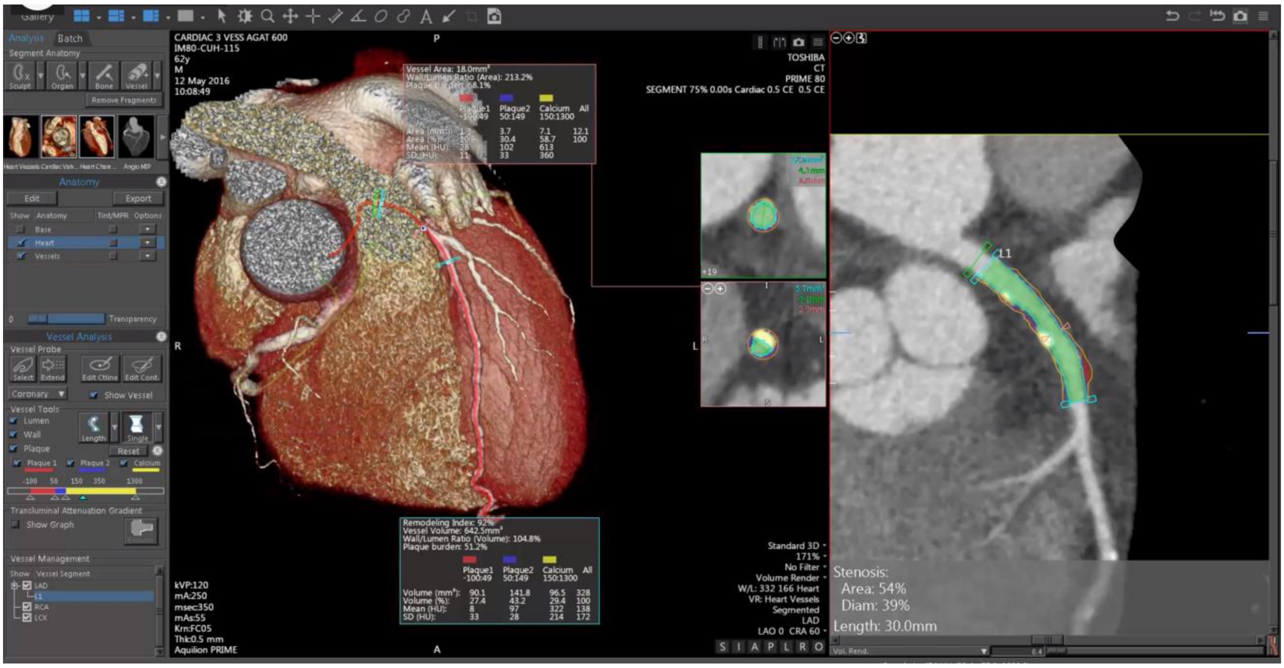

SUPPLEMENT 3

RADIATION EXPOSURE

Instead of using individual patient dosimeters, dose-length product (DLP) dosimetry metrics individually reported from each CCTA and the dose area product (DAP) from each PCI were used. For CCTA, the dose-length product, defined as the total radiation energy absorbed by the patient's body, was measured in mSv/mGy·cm. The effective radiation dose was calculated as the product of the dose-length product times a conversion coefficient for the chest (K = 0.014 mSv/mGy·cm).

The DLP was 741±198 mGy/cm, which is equivalent to 10.4±2.8 mSv. For the PCI, the effective radiation dose was calculated by multiplying the dose-area product by a conversion factor (K=0.21 mSv/mGy·cm^2) for lateral and posteroanterior radiation exposure in the chest area. The DAP was 782±209 mGy/cm, which is equivalent to 13.2±3.5 mSv.

SUPPLEMENT 4

OCT IMAGE ACQUISITION

Intravascular OCT of the entire target vessel was performed before the PCI. After intracoronary administration of nitroglycerin (100–300 microg), a commercially available frequency-domain OCT system (ILUMIEN/ILUMIEN OPTIS OCT Intravascular Imaging System, St. Jude Medical, St. Paul, MN, USA) was advanced to the distal part of the artery behind the more distal calcified plaque. OCT pullback was performed at 20 mm/s with continuous injection of contrast medium through the guiding catheter. The raw OCT data were anonymized and digitally stored in an offline review system (St. Jude Medical™) for subsequent analysis.

OCT DATA ANALYSIS

The OCT image analysis was performed by two experienced observers who were blinded to the clinical and the CTA data using previously established criteria for OCT plaque characterization. The vessel with the culprit lesion identified by angiography, with the aid of clinical data as necessary, was analyzed by OCT. The other coronary arteries were explored with OCT if they had a significant calcified plaque by CCTA. In order to ensure that identical coronary areas were assessed by CCTA and OCT, we identified the location of a plaque detected using OCT by measurement of the distance from a specific landmark such as the coronary ostium or branches.

First of all, each calcified plaque was classified as vulnerable or not. The segmentation was carried out on the thin-cap fibroatheroma (TCFA) and/or the thrombus. The fibrous cap thickness was measured three times at its thinnest part, and the average value was calculated. A TCFA was defined as a lipid-rich plaque with a cap thickness of ≤ 65 mm. A thrombus was defined as an irregular mass with high or low backscattering protruding into the lumen. The thrombus type was further classified as either red or white. A red thrombus was defined as a high backscattering protrusion with signal-free shadowing on OCT. A white thrombus was defined as a signal-rich, low backscattering projection. Calcified plaques with a TCFA and/or a thrombus were considered to be vulnerable.

In order to make a comparison with CCTA analysis of plaques, the presence of lipid, TCFA, plaque rupture, calcification, thrombus, macrophage accumulation, cholesterol crystals, and microchannels on OCT images was evaluated within a 10 mm segment (5 mm proximal to 5 mm distal to each calcified lesion at the smallest lumen cross-sectional area), in accordance with previous studies. If the two observers had discordant diagnoses, a consensus was obtained

using repeated offline readings. When lipid, characterized by signal-poor regions with diffuse borders, was present for > 90° in any cross-sectional images, the plaque was considered to be lipid-rich. For each plaque, the lipid core length was defined as the longitudinal length of contiguous cross-sections that fulfilled the definition of lipid-rich plaque. Calcification was defined as a well-delineated signal-poor region with sharp borders. Macrophage accumulation was defined as bright spots with high OCT backscattering signal variances. A microchannel was defined as a black hole or tubular structure within a plaque observed on ≥ 3 consecutive cross-sectional images. A cholesterol crystal was defined as a thin linear region of high signal intensity within a lipid plaque. Plaque rupture was defined as intimal interruption and cavity formation in a plaque. Healed plaque rupture was detected as a landmark with multiple tissue layers of different optical densities overlying a large lipid core in the presence or absence of calcification. Plaque erosion was defined as an irregular luminal surface, and no evidence of cap rupture evaluated in multiple adjacent frames.

Interobserver and intraobserver variability were assessed based on evaluation of all of the images by two independent readers and by the same reader at two separate time points, respectively.
